# Uptake and localisation of mTHPC (Foscan®) and its^14^C-labelled form in normal and tumour tissues of the hamster squamous cell carcinoma model: a comparative study

**DOI:** 10.1038/sj.bjc.6600651

**Published:** 2002-12-02

**Authors:** S Andrejevic Blant, T M Glanzmann, J-P Ballini, G Wagnières, H van den Bergh, P Monnier

**Affiliations:** Institute of Pathology, CHUV-Hospital, Bugnon 21, CH-1011 Lausanne, Switzerland; Institute of Environmental Engineering, EPFL, CH-1005 Lausanne, Switzerland; Department of Otolaryngology Head and Neck Surgery-CHUV Hospital CH-1011 Lausanne, Switzerland

**Keywords:** mTHPC fluorescence spectroscopy, fluorescence microscopy, photosensitiser, ^14^C-meta(tetrahydroxyphenyl)chlorin, pharmacokinetics, squamous cell carcinoma

## Abstract

The aim of this study was to evaluate the pharmacokinetics of meta(tetrahydroxyphenyl)chlorin (mTHPC) on different tissues of interest in a hamster tumour model and to confirm our earlier animal studies on semi-quantitative fluorescence microscopy. The results obtained by three different evaluation methods were compared: *in vivo* spectrofluorometry, *ex vivo* fluorescence microscopy and chemical extraction of ^14^C-labelled mTHPC. Following intracardiac injection of 0.5 mg kg^−1^ mTHPC, groups of five tumour-bearing animals were used for *in* *situ* light-induced fluorescence spectroscopy. Afterwards, the biopsies were taken and snap frozen for fluorescence microscopy. The presence of radioactivity in serum and tissues was determined after chemical digestion in scintillation fluid using a scintillation counter. For each analysed tissue, a good correlation was observed between the three evaluation methods. The highest fluorescence intensity and quantities of mTHPC were observed between 12 and 24 h in liver, kidney, serum, vascular endothelium and advanced neoplasia. The majority of mTHPC was found at around 48 h in smooth muscle and at 96 h in healthy cheek pouch mucosa and early malignant lesions. The lowest level of mTHPC was noted in striated muscle at all times. No selectivity in dye localisation was observed between early squamous cell carcinoma and healthy mucosa. Soon after the injection, a significant selectivity was noted for advanced squamous cell carcinoma as compared to healthy cheek pouch mucosa or striated muscle. A significant difference in mTHPC localisation and quantity was also observed between striated and smooth muscle during the first 48 h following the injection. Finally, this study demonstrated the usefulness of non-invasive *in situ* spectroscopic measurements to be performed systematically prior to photodynamic therapy as a real-time monitoring for each treated patient in order to individualise and adapt the light dosimetry and avoid over or under treatments.

*British Journal of Cancer* (2002) **87**, 1470–1478. doi:10.1038/sj.bjc.6600651 www.bjcancer.com

© 2002 Cancer Research UK

## 

Photodynamic therapy (PDT) involves the administration of a photosensitiser (PS) and the local illumination of the target with the light of a wavelength corresponding to an absorption peak of the administered drug. The absorption of the light energy by a PS induces a photochemical reaction leading to the damage of the illuminated tissue (Gibson *et al*, 1994). Over the past two decades, this minimally invasive treatment modality has continued to be evaluated for its effectiveness in eradication of malignant (Biel, 1995; Grosjean *et al*, 1996a; Ris *et al*, 1996) and non-malignant lesions (Hsiang *et al*, 1993; Haimovici *et al*, 1997; Trauner *et al*, 1998). Amount and time-dependent localisation of the photosensitiser in various tissues play a key role in the mechanism of destruction induced by PDT. The knowledge of pharmacokinetics and tissue affinity of the PS helps to give further insight into fundamental phototoxicity mechanisms such as vascular *vs* cellular damage and their relative importance to tissue destruction. Photodynamic therapy with meta(tetrahydroxyphenyl)chlorin (mTHPC) has been shown in clinical trials to be a safe and effective treatment of early malignancy in the upper aero-digestive tract, the bronchi and the oesophagus (Grosjean *et al*, 1996b; Savary *et al*, 1997). Numerous studies have shown that mTHPC, like other anti-cancer drugs (Mcloed and We, 1995), has high inter-patient variability for accumulation of the drug in normal or malignant tissues (Braichotte *et al*, 1996; Glanzmann *et al*, 1998). This variability is probably a major factor in the large range of tissue responses to PDT in different patients who have undergone treatment under identical conditions (e.g., drug dose, light dose, light dose rate, time delay after drug administration). The tissue response to PDT could be apparently improved by taking into account the real-time PS concentration determined by light-induced fluorescence spectroscopy (LIFS). This non-invasive *in situ* technique enables the treatment conditions to be adapted and the light dose to be individualised for each treated patient to avoid over or under treatment (Wagnières *et al*, 1998). To clarify these *in vivo* data, and to introduce LIFS systematically *in situ* before each PDT, one of the main goals of this study was to confirm and correlate the data obtained by LIFS with two other evaluation methods.

Our earlier animal studies on semi-quantitative fluorescence microscopy (FM) (Andrejevic *et al*, 1996; Andrejevic Blant *et al*, 2000) have shown the much lower uptake and retention of mTHPC in striated muscle as compared to normal oral mucosa, squamous cell carcinoma (SCC) and smooth muscle. These findings are of major importance in clinical PDT for treating SCCs with unfavourable localisations safely, such as the base of the tongue or the oesophagus. Another goal of this study was to compare our earlier data on FM to those obtained by *in vivo* spectrofluorometry and chemical extraction of ^14^C-labelled mTHPC in the hamster squamous cell carcinoma model. The pharmacokinetic pathway and the tissue affinity of mTHPC, determined by three different evaluation methods are of interest for a better understanding of the mechanisms of distribution, retention and tissue damage after mTHPC-PDT.

## MATERIALS AND METHODS

### Animal model

**Figure 1:**
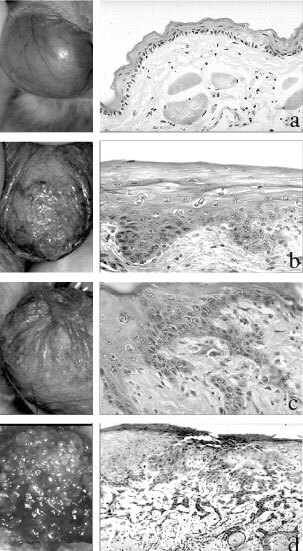
Macroscopic appearance on the *left side* and histology on the *right side* of the hamster cheek pouch during various steps of squamous cell carcinogenesis. (**A**) Smooth and uniform surface of the healthy cheek pouch with thin mucosa; corresponding histology showing the top 3–4 layers of squamous epithelial cells (**E**) including well-delimited basal membrane (BM); lamina propria (LP) containing stromal cells, fibro-connective tissue and blood vessels and underlying striated muscle (SM). (**B**) Early dysplastic changes varying from low- to high-grade dysplasia appear between 6 and 9 weeks after DMBA application. Macroscopically, the cheek pouch mucosa is more-or-less thick, showing erythroleukoplastic changes. Histologically, this corresponds to an abnormal proliferation of epithelial cells with large nuclei, abundant cytoplasm and frequent mitotic figures. BM is less well delimited. No isolated tumour cells are visible in the lamina propria. (**C**) About 10 weeks after DMBA application, erythroleucoplastic changes, irregularity and thickening of the mucosa become much more severe. Histologically, besides the cytonuclear abnormalities, a small island of isolated tumour cells appears in the lamina propria. This stage corresponds to μ-invasive carcinoma often associated with slight peritumoural inflammation. (**D**) At 16 weeks following DMBA application, the bulky formation appears on the painted cheek pouch surface, corresponding to more-or-less well-differentiated SCC. At this stage, a tumour shows a highly infiltrative pattern and large tumour nests invade the lamina propria and striated muscle. The lesions are highly vascularised and tumour-associate inflammation is abundant. Original magnification ×10 (**A**,**D**) and ×20 (**B**,**C**).

Female Syrian hamsters (BRL, Fuellinsdorf, CH) weighing 140–160 g, housed at room temperature with 12 h light/dark cycle were used in this study. Free access to food and drinking water was allowed throughout the experiments. The animals were divided into two groups (early and advanced SCC) prior to chemically induced carcinogenesis. Early and advanced SCCs were chemically induced by topical application of 5% oily DMBA (Sigma Chemicals, Co., St. Louis, MO, USA) solution in the hamster's left cheek pouch mucosa three times weekly during 10–12 weeks (early SCC) and 14–16 weeks (advanced SCC), respectively. The contralateral buccal pouch mucosa, which was not painted with DMBA, served as the healthy mucosa control (Figure 1A

). The animals were regularly checked to document the development of neoplastic changes. Up to five tumour-bearing animals were used for each analysed time point. *In vivo* measurements were performed under intraperitoneal anaesthesia (Ketalar 150 mg kg^−1^ and Xylesine 15 mg kg^−1^). After *in situ* measurements, the animals were sacrificed and the tissue samples were collected and prepared for fluorescence microscopy analysis. The animals that received labelled mTHPC were sacrificed at the same points in time as those for LIFS and FM, and tissues were sampled for quantification of ^14^C-mTHPC. All experiments were carried out following the recommendation of guidelines for the welfare of animals in experimental neoplasia (Workman *et al*, 1998) and in accordance with the protocol approved by the Experimental Animal Ethics Committee of the CHUV Hospital.

### Definition and staging

‘Early stage’ SCCs were considered to be those that were either *in situ* (Tis), i.e. intra-epithelial with no invasion of the basement membrane, or micro-invasive (μ-invasive SCC), i.e. with no invasion beyond the lamina propria of the cheek pouch mucosa. Between 6 and 9 weeks from the first DMBA application, different degrees of epithelial dysplasia were observed (Figure 1B), which evolved to Tis and μ-invasive SCCs (Figure 1C) between 10 and 12 weeks.

For advanced neoplastic lesions (Figure 1D), the tumour growth was carefully observed after 12 weeks and documented weekly by measurements of three axes (L×W×H). The animals were sacrificed when tumours had reached a diameter no greater than 8–10 mm, corresponding to a volume of 350 (±100) mm^3^. All tumours were well vascularised and free of necrosis at this size. Macroscopic and histopathologic aspects of the normal hamster pouch mucosa and steps of SC carcinogenesis from early to advanced SCC are shown in Figure 1A–D (see legend to Figure 1). The histopathologic changes occurring during DMBA-induced chemical carcinogenesis were highly reproducible in different groups of animals. Macroscopically and microscopically, they were very close to the human forms of ‘early stage’ (dysplasia, carcinoma *in situ* or μ-invasive carcinoma) and invasive SCCs in the upper digestive tract and oesophagus (Andrejevic *et al*, 1996).

### Photosensitiser

The mTHPC, now known under the trade name Foscan®, as well as its ^14^C-labelled form, were kindly supplied as a lyophilised powder by Quanta-Nova (Guildford, UK). They were freshly prepared before use by dissolving them in a mixture of 30% (v v^−1^) polyethylene glycol 400, 20% (v v^−1^) ethanol and 50% (v v^−1^) H_2_O. mTHPC, ^14^C-labelled in the meso positions, was custom-synthesised by American Radiolabeled Inc. (St. Louis, MO, USA) with a specific activity of 74 μCi mmol^−1^ and a purity at 97%.

### Comparative pharmacokinetics study

After intracardiac injection of 0.5 mg kg^−1^ mTHPC or ^14^C-mTHPC, groups of five tumour-bearing animals were sacrificed at various points of time ranging from 4 h to 8 days. Before sacrifice, non-invasive *in situ* LIFS measurements were performed in animals injected with non-labelled mTHPC on various organs such as the liver, kidney, healthy cheek pouch mucosa, early and advanced SCC, striated (skeletal) and smooth muscle (uterus). Then the blood was collected, the serum was separated by centrifugation and LIFS measurements of serum were carried out. Following this step, the various tissue samples were fast frozen and prepared for fluorescence microscopy analysis. The animals injected with ^14^C-mTHPC were sacrificed at the same times and similar tissues were sampled for ^14^C-mTHPC quantification.

#### *In vivo* pharmacokinetic measurements by LIFS

Non-invasive *in vivo* LIFS was performed with an optical fibre-based spectrofluorometer described previously (Zellweger *et al*, 1999). Briefly, the excitation light of a high-pressure xenon lamp was spectrally filtered by a spectrograph and coupled into an optical fibre. This fibre was held in gentle contact with the tissue under investigation. The emitted fluorescence of the tissue was collected by the same fibre, separated from the excitation path by a dichroic filter and spectrally dispersed by a second spectrograph. The fluorescence spectrum was recorded by a Peltier-cooled CCD camera and processed by a microcomputer. A fluorescence standard (Rhodamine B, 1 μM in water) was used to calibrate the intensity of the tissue fluorescence for changes in excitation intensity and light collection efficiency of the optical system. The absorption peak of mTHPC at 420 nm was chosen as the excitation wavelength in order to be sensitive essentially to the uppermost layers of mucosa. The spectrum of the tissue autofluorescence was subtracted from the recorded spectrum. The height of the emission peak at 652 nm of the mTHPC fluorescence was used as a relative measure for the PS quantification. The fluorescence intensity obtained in this way was reported in the corresponding figures in relative units for *in vivo* fluorescence (LIFS) (r.u. for 2a,3a,4a,5a) and for *ex vivo* fluorescence microscopy (FM) (r.u. for 2c,3c,4c,5c). For each tissue of interest, we took 5–10 spectra by slightly displacing the fibre on the tissue in order to reduce the influence of local inhomogeneities. The results were presented as mean values of these measurements and are reported together with the corresponding standard deviations.

#### *Ex vivo* fluorescence microscopy-FM

The biopsies taken were fast frozen by contact with an isopentane slush and stored at −70°C prior to use. Tissue sections were prepared in the dark to avoid photobleaching. Five consecutive non-stained 5-μm thick tissue sections mounted on clean glass slides were prepared from each sample. From each frozen section, three images were recorded at three different parts of the slice to avoid photobleaching. The autofluorescence background substraction procedure was standardised on tissue slices from five uninjected animals. The relative fluorescence intensity was analysed using the public domain NIH Image 1.62 program. After recording the fluorescence images, the same slices were carefully removed and stained with haematoxilin and eosin (HE). An HE image was recorded at an identical position. The HE image was compared with the fluorescence image in order to determine the exact histological localisation of the PS fluorescence. We employed an Olympus BH-2 epifluorescence microscope with a filtered 100 W mercury lamp as the excitation light source. Fluorescence images were detected with a cooled slow-scan 16-bit CCD camera (LEEV P86231, Wright Instruments, Endfield, UK). For excitation, an interference band pass filter 420DF30 and a dichroic mirror at 470 nm were used. A long pass filter RG 630 was used to record the fluorescence of mTHPC. An interference band pass filter 560DF40 was used to record the tissue autofluorescence. The CCD camera was operated with an excitation shutter to avoid photobleaching. The system was controlled by an IBM-PC computer using the AT1 software (Wright Instruments), which processes 16-bit images. The localisation and intensity of the dye fluorescence was ascertained by subtracting the autofluorescence from the fluorescence image. Flat field correction was done using a fluorescent reference sample. The fluorescence intensity obtained in this way was reported in relative units (r.u.) in the corresponding figures.

#### Chemical extraction of ^14^C-labelled mTHPC

The time-dependent presence of radioactivity in serum and various tissues was determined after administration of 0.5 mg kg^−1^ mTHPC containing 55 μCi of ^14^C-labelled mTHPC. Specific activity of ^14^C-mTHPC was calculated before the experiment. Each animal received 5 μCi of ^14^C per 100 g of body weight. Animals were sacrificed at the same points of time, as in the case of LIFS and FM. The blood was collected, and the serum was separated after centrifugation at 4000 **g** for 10 min. The various healthy tissues of interest and tumours were surgically excised, rinsed in saline, blotted dry and homogenised with scissors. The samples were weighed and digested in 0.2 N NaOH (1 ml per 200 mg of homogenate) by shaking at 50°C overnight. After digestion, the total radioactive contents of each sample were determined in 4 ml of scintillation liquid using a liquid scintillation counter and calculated as a per cent of total injected dose. Data were expressed in μg of extracted ^14^C-labelled mTHPC g^−1^ of tissue. Background radioactivity was measured using corresponding samples from three animals that were administered unlabelled mTHPC. It never exceeded 23 d.p.m. g^−1^ of tissue and was similar to that of scintillation liquid alone.

### Statistical analysis

The significance of fluorescence intensities at various points of time, measured either by *in vivo* or *ex vivo* methods, was determined according to a non-parametric Mann–Whitney *U*-test (α⩽0.05). The results were presented as mean values of these measurements and were reported together with the corresponding standard deviations (s.d.). For extraction data, the mean concentration of five samples and their s.d. were calculated for each time point. The data points were fitted with the following function (y=a exp (-bt) (1-exp (-ct))) for visual support.

## RESULTS

**Figure 2:**
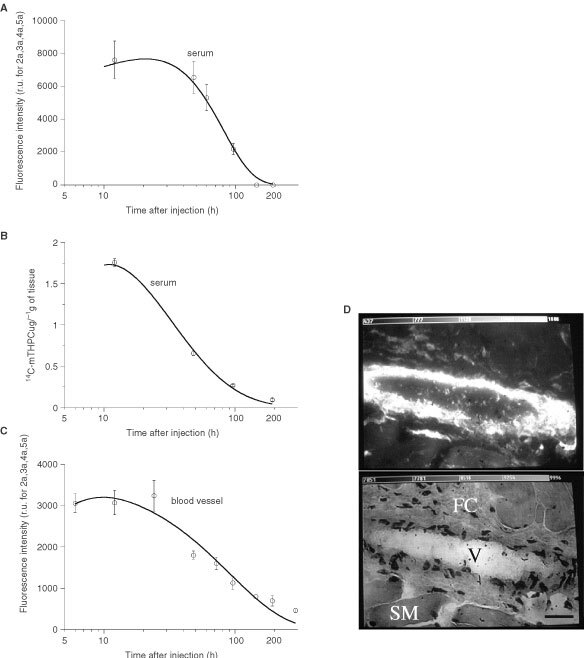
The comparative pharmacokinetics of mTHPC in serum and vascular endothelium by means of *in situ* LIFS (**A**), extraction of ^14^C-labelled mTHPC (**B**) and *ex vivo* FM (**C**). The fluorescence photomicrograph and corresponding HE staining of the blood vessel wall 48 h after mTHPC administration shows a much higher fluorescence intensity in blood vessel (V) endothelium as in adjacent structures such as fibro-connective tissue (FC) of lamina propria and striated muscle (SM) (**D**). Scale: the bar in the right bottom corner represents 20 μm.

The relative fluorescence intensities or quantities of mTHPC measured by the three methods showed a heterogeneous PS distribution in different tissues at various times after administration. The autofluorescence level recorded on different tissues using LIFS or FM was fairly comparable between different animals. It ranged from 300 r.u. for striated muscle and 700 r.u. for fibro-elastic and collagen tissue of the lamina propria (data not shown). The mTHPC fluorescence and tissue distribution were within the same order of magnitude at all measured points. Figures 2

,3

**Figure 3:**
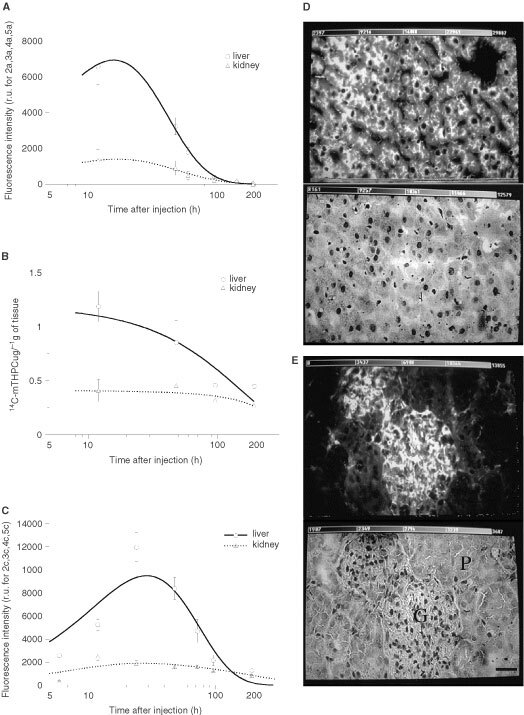
The comparative pharmacokinetics of mTHPC in the liver and kidney using *in situ* LIFS (**A**), extraction of ^14^C-labelled mTHPC (**B**) and *ex vivo* FM (**C**). Fluorescence photomicrographs and corresponding HE stains show the high fluorescence intensity, uniform in liver parenchyma (**D**) and kidney glomerulus (white areas), while a low dye amount was observed in proximal and distal tubes of kidney parenchyma (dark area) soon (24 h) after mTHPC administration (**E**). Scale: the bar in the right bottom corner represents 20 μm.

,4

**Figure 4:**
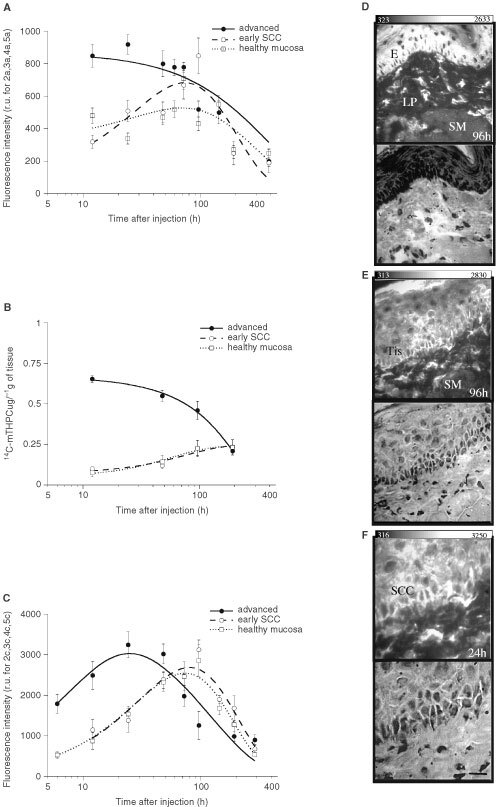
The comparative pharmacokinetics of mTHPC in healthy mucosa, early and advanced SCC observed by *in situ* LIFS (**A**), extraction of ^14^C-labelled mTHPC (**B**) and *ex vivo* FM (**C**). The photomicrographs and corresponding HE stains of healthy hamster mucosa (**D**), early (**E**) and advanced SCC (**F**) show high fluorescence intensity in the epithelial layers in both normal or tumoural epithelium (E), while adjacent structures such as underlying lamina propria (LP) and striated muscle (SM) show a weak fluorescence intensity. Scale: the bar in the right bottom corner represents 20 μm.

,5

**Figure 5:**
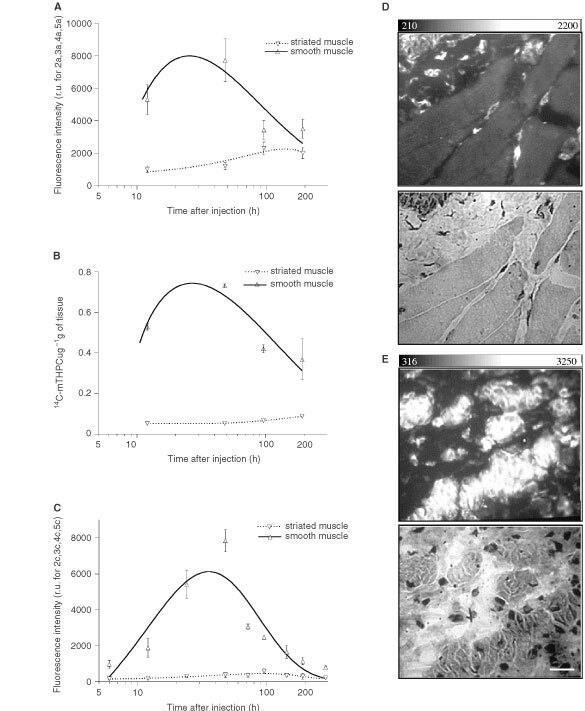
The comparative time-dependent fluorescence and quantity of mTHPC in striated and smooth muscle observed by *in situ* LIFS (**A**), extraction of ^14^C-labelled mTHPC (**B**) and *ex vivo* FM (**C**). Fluorescence photomicrographs and corresponding HE stains of striated (**D**) and smooth muscles (**E**) at 72 h after dye administration show a weak and negligible fluorescence intensity in striated muscle, while a very high dye level was noted in the smooth muscle. Scale: the bar in the right bottom corner represents 20 μm.

display the results of three evaluation methods in different organs and tissues of interest.

Figure 2 illustrates a comparison of fluorescence quantities measured in serum by means of LIFS (Figure 2A), chemical extraction of ^14^C-labelled mTHPC (Figure 2B), as well as the relative fluorescence intensity recorded on normal blood vessel endothelium by FM (Figure 2C). The highest mTHPC level in endothelial cells of the blood vessels between 12 h and 1 day after dye administration was observed by FM. The position of this maximum was compatible with the measurements obtained in the serum using the two other mTHPC assessment techniques. After 1 day, the dye level decreased rapidly either in the serum or in the vascular endothelium with an elimination lifetime between 2 and 3 days. It returned to background level at about 8 days after administration. Fluorescence micrograph and corresponding HE staining of the blood vessel wall 2 days after mTHPC administration are shown in Figure 2D. A much higher fluorescence intensity was observed in blood vessel endothelium than in adjacent structures such as fibro-connective tissue of the lamina propria and striated muscle.

The pharmacokinetics of mTHPC in the liver and kidney presented similarities to those seen in the vasculature. Whatever the method used, the highest dye amount was observed in liver parenchyma and the excretory part of the kidney (glomerulus) between 12 and 24 h after dye administration. The dye amount decreased almost to the background level 96 h after administration (Figure 3A–C).

The fluorescence photomicrographs and the corresponding HE stains in Figure 3D and E present the fluorescence intensities in the liver and kidney observed at 24 h after mTHPC administration. The advantage of FM over the other two methods was that the PS could be localised at the cellular level. As shown in Figure 3E, a high mTHPC amount was observed in kidney glomerulus (white area), and weak fluorescence was recorded in proximal and distal tubules of kidney parenchyma (dark area). This observation suggests a rapid elimination of the dye from the excretory part of the kidney. In order to compare the FM study in kidney to the two other methods used, the mean fluorescence intensity of the whole kidney, including the glomerulus (excretory compartment) and parenchyma (collecting compartment), was plotted against time in Figure 3C.

A fairly similar kinetic pathway and no significant selectivity in mTHPC fluorescence or quantity between healthy mucosa and early SCC were observed by each of the three methods used (Figure 4). However, following a short delay after dye administration, a significant selectivity (α⩽0.05) was noted between advanced SCC and healthy mucosa. (deleted) (Figure 4A–C). The photomicrographs and corresponding HE stains of healthy hamster mucosa (Figure 4D), early (Figure 4E) and advanced SCC (Figure 4F) recorded at 1 (advanced SCC) and 4 days (early SCC and healthy mucosa) after mTHPC administration show a high fluorescence in either normal or tumoural epithelium. By contrast, weak fluorescence intensity was observed in adjacent structures such as underlying lamina propria, stromal endothelial cells and striated muscle. The homogenous fluorescence in the cytoplasm, surrounding the dark area corresponding to the nuclei, suggests an intracellular localisation of mTHPC.

All three methods used in this study showed low time-dependent mTHPC fluorescence and a negligible quantity in striated muscle (Figure 5A–C). By contrast, a significantly higher (α⩽0.05) amount of fluorescence or quantity was noted in smooth muscle with a peak around 2 days after dye administration. Figure 5D,E show fluorescence photomicrographs and corresponding HE stains of striated and smooth muscle 3 days after mTHPC administration. Weak and negligible fluorescence intensity was observed in striated muscle, while a very high dye level was noted in smooth muscle. Furthermore, apart from serum and liver, the maximum mTHPC signal in smooth muscle between 2 and 3 days after administration was significantly higher than any signal seen in other quantified tissues.

## DISCUSSION

The present comparative study using LIFS and chemical extraction of ^14^C-labelled mTHPC gives an overview and confirms the pharmacokinetic data in several tissues of interest relevant for clinical PDT applications. The results presented here are in agreement with the FM study reported previously by our group (Andrejevic Blant *et al*, 1998, 2000). At a short delay (>2 days) after injection, the mTHPC was found in the liver, kidney, vasculature (either vascular bed or serum) and advanced SCC. At longer delays, mTHPC was mainly observed in the epithelial layer of the oral mucosa, and it showed a particularly high affinity for smooth muscle.

In contrast to the high mTHPC affinity for smooth muscle, very low dye fluorescence was noted in striated muscle, stromal endothelial cells and fibro-connective tissue. One possible explanation for this finding, observed by means of three different evaluation approaches, could be a different embryogenic origin of these tissues, and/or lipophilic properties and molecular structure of their cytoplasmic membrane. The fibro-connective tissue and striated muscle develops from endodermis, while smooth muscles arise from the ectodermal germ layer. In addition, the nerve supply of these two types of muscle is quite different. Motor nerves supply the striated muscle, while a smooth muscle receives its innervations from the autonomic nervous system by neuromuscular synapses on the membrane's surface. The quantity and/or disposition of lypophilic and hydrophilic surface-cell-membrane molecules, ‘neurotransmitters’, are probably responsible for different membrane charges and their consecutive affinity for various PS. From a clinical point of view, the low mTHPC level in striated muscle provides the opportunity to develop interstitial PDT as a minimally invasive treatment modality for more advanced SCC of unfavourable localisation in the oral cavity or pharynx such as the base of the tongue. It is also pertinent to note that PDT treatment of the middle and lower oesophagus, which contains mostly smooth muscle in its wall, should be treated with extreme caution in order to avoid complications such as perforation and fistula.

None of the methods used here permitted to find a significant time-dependent selectivity between early SC malignancies and healthy mucosa. However, at a short delay after mTHPC administration, a significant fluorescence contrast (α⩽0.05) was observed between advanced SCC and healthy mucosa. Several histopathological features observed in advanced SCC should explain in part this selectivity. As with many human or animal tumours, the DMBA-induced advanced SCC hamster model presents a large number of tumour vessels as compared to healthy mucosa, as well as a more-or-less severe peritumoural inflammatory reaction (Lurie *et al*, 1983). In addition, the ultrastructural studies in human and experimental animals have demonstrated an increased permeability of tumour vessels (Feng *et al*, 2002).

All of these features should explain a much higher accumulation of mTHPC shortly after administration in invasive SCC as compared to normal tissue or early SCC. In our study, this was supported by the mTHPC distribution in serum and advanced SCC as shown in Figures 2 and 4. One can note that the half-life of mTHPC in serum is between 2 and 3 days after injection (Figure 2), whatever the method used, and that these points in time coincide with the maximal mTHPC level in advanced SCC (Figure 4).

The results reported in this study are in good agreement with results of biodistribution and pharmacokinetics of ^14^C-labelled mTHPC in other rodent models (Whelpton *et al*, 1996; Obwegeser *et al*, 1998). Numerous studies on *ex vivo* or *in vivo* quantification of other first and second-generation photosensitisers such as phthalocyanines (Lee *et al*, 2001), photofrin (Gomer and Dougherty, 1979), or aminolevulinic-acid induced PPIX (Obwegeser *et al*, 1998), using similar evaluation methods are also in agreement with the results reported in our study, especially investigating the localisation of the PS in smooth muscle.

It is important to note that measurements obtained by either LIFS or fluorescence microscopy may also be subject to errors from environmental factors that affect the fluorescence yield. For both methods used, the fluorochrome environment was the same for all animals at various time intervals. This effect should thus not affect our results as all of our samples, whether *in vivo* or *ex vivo,* were processed following the same protocol.

It is also well known that *in situ* LIFS measurements are highly sensitive to the tissue type, as mentioned by several authors (Pouge and Burke, 1998; Wagnières *et al*, 1998; Westermann *et al*, 1998). In assessing the quantification of fluorescence *in situ,* it is necessary to consider three major points: (a) the tissue's optical properties of the excitation and emission light; (b) the fluorescence quantum yield of the PS used in its environment and (c) the geometry of the measurements. Indeed, if the fluorescence quantum yield of the PS changes with the environment of the latter, then the fluorescence signal and the corresponding PS concentration are no longer related in the same manner. Some of these points have been studied in detail by our group using mTHPC and its PEG-coupled form on a human xenograf colon carcinoma model. A good correlation between LIFS, quantification of radioactivity and improvement of *in vivo* tumour localisation reported by Westermann *et al* (1998) are in good agreement with our study.

The measured values obtained by LIFS and FM as well as quantities of extracted mTHPC in different analysed tissues allow us to conclude that there is a highly reproducible ‘quantitative correlation’ between different tissues of interest. The ratios of nearly 3 : 1 between advanced SCC and normal mucosa and 7:1 between smooth and striated muscle were observed by all three methods.

One of the major unresolved issues in PDT research is the development of optimal dosimetry tools useful for photosensitiser quantification *in situ*. Our study has shown that any of the three methods should be suitable either when used together or separately for preclinical screening of new generation PS, as long as the therapeutic dose is a function of the mTHPC fluorescence or concentration.

Two main conclusions resulting from this study are that the LIFS measurements are confirmed by absolute concentration of ^14^C-labelled mTHPC, and that *ex vivo* FM measurements are in agreement with *in vivo* LIFS data. This permits us to develop concept of ‘validation’ of *ex vivo* techniques by *in vivo* LIFS measurements. The observed correlations support the hypothesis that non-invasive *in vivo* LIFS provide real-time information useful for adapting light dosimetry for each patient treated by PDT, in order to avoid under or over treatment and undesirable side effects. Therefore, LIFS for individualised *in vivo* dosimetry is a promising *in situ* technique, which needs to be improved upon by evaluating a large number of therapeutic results.

We hope that individualised dosimetry will be relevant for a better understanding of the distribution and kinetics of new photosensitisers and thus will contribute to increasing the efficacy of clinical trials. This assumes that the best therapeutic results will be obtained, side effects will be diminished, and complications will be avoided.

## References

[bib1] Andrejevic BlantSBalliniJvan den BerghHFontollietCWagnièresGMonnierP2000 Time-dependent biodistribution of tetra(m-hydroxyphenyl)chlorin and benzoporphyrin derivative monoacid ring A in the hamster model: Comparative fluorescence microscopy study Photochem Photobiol 71 333340 10732452 10.1562/0031-8655(2000)071<0333:TDBOTM>2.0.CO;2

[bib2] Andrejevic BlantSWoodtliAWagnieresGFontollietCVan den BerghHMonnierP1998 Interstitial photodynamic therapy with tetra(m-hydroxyphenyl)chlorin: tumour versus striated muscle damage Intl J Radiat Oncol Biol Phys 42 403412 10.1016/s0360-3016(98)00221-1 9788423

[bib3] AndrejevicSSavaryJFMonnierPFontollietCBraichotteDWagnieresGvan den BerghH1996 Measurements by fluorescence microscopy of the time-dependent distribution of meso-tetra-hydroxyphenylchlorin in healthy tissues and chemically induced ‘early’ squamous cell carcinoma of the Syrian hamster cheek pouch J Photochem Photobiol B Biol 36 143151 10.1016/s1011-1344(96)07362-9 9002251

[bib4] BielMA1995 Photodynamic therapy of head and neck cancers Sem Surg Oncol 11 355359 10.1002/ssu.2980110505 7569557

[bib5] BraichotteDRSavaryJFMonnierPvan den BerghHE1996 Optimizing light dosimetry in photodynamic therapy of early stage carcinomas of the esophagus using fluorescence spectroscopy Lasers Surg Med 19 340346 8923430 10.1002/(SICI)1096-9101(1996)19:3<340::AID-LSM10>3.0.CO;2-8

[bib6] FengDNagyJADvorakHFDvorakAM2002 Ultrastructural studies define soluble macromolecular, particulate, and cellular transendothelial cell pathways in venules, lymphatic vessels and tumour-associated microvessels in man and animals Micro Res Tech 57 289326 10.1002/jemt.10087 12112440

[bib7] GibsonSLFosterTHFeinsRHRaubertasRFFallonMAHilfR1994 Effects of photodynamic therapy on xenografts of human mesothelioma and rat mammary carcinoma in nude mice Br J Cancer 69 473481 8123476 10.1038/bjc.1994.86 PMC1968871

[bib8] GlanzmannTHadjurCZellwegerMGrosieanPForrerMBalliniJMonnierPvan den BerghHLimCWagnieresG1998 Pharmacokinetics of tetra(m-hydroxyphenyl)chlorin in human plasma and individualised light dosimetry in photodynamic therapy Photochem Photobiol 67 596602 9613244

[bib9] GomerCDoughertyT1979 Determination of 3H and 14C hematoporphyrin derivative distribution in malignant and normal tissue Cancer Res 39 146151 761185

[bib10] GrosjeanPSavaryJMizeretJWagnièresGWoodtliATheumannJFontollietCvan den BergHMonnierP1996a Photodynamic therapy for cancer of the upper aerodigestive tract using tetra(m-hydroxyphenyl)chlorin J Clin Care Med Surg 14 281287 10.1089/clm.1996.14.281 9612194

[bib11] GrosjeanPSavaryJWagnièresGMizeretJWoodtliATheumannJFontollietCvan den BergHMonnierP1996b Tetra(m-hydroxyphenyl)chlorin clinical photodynamic therapy of early bronchial and esophageal cancer Lasers Med Sci 11 227235

[bib12] HaimoviciRKramerMMillerJHasanTFlotteTSchomackerKGragoudasE1997 Localisation of lipoprotein-delivered benzoporphyrin derivative in the rabbit eye Curr Eye Res 16 8390 9068937 10.1076/ceyr.16.2.83.5088

[bib13] HsiangYNCrespoMTRichterAMJainAKFragosoMLevyJG1993 In vitro and in vivo uptake of benzoporphyrin derivative into human and miniswine atherosclerotic plaque Photochem Photobiol 57 670674 8506397 10.1111/j.1751-1097.1993.tb02935.x

[bib14] LurieATatematsuMNakatsukaTRippeyRItoN1983 Anatomical and functional vascular changes in hamster cheek pouch during carcinogenesis induced by 7,12-dymethylbenz(a)anthracene Cancer Res 43 59865994 6416672

[bib15] LeeCPougeBStrawbridgeRMoodieKBartholomewLBurkeGJack HoopesP2001 Comparison of photosensitiser (AlPcS2) quantification techniques: in situ fluorescence microsampling versus tissue chemical extraction Photochem Photobiol 74 453460 11594060 10.1562/0031-8655(2001)074<0453:copaqt>2.0.co;2

[bib16] McLoedHWeE1995 Clinical pharmacokinetics of anticancer drugsInHandbook of Pharmacokinetics/Pharmacodynamics correlationDerendorf H, Hochhaus G (eds)pp 389–414Boca Raton, Florida: CRC Press

[bib17] ObwegeserAJakoberRKostronH1998 Uptake and kinetics of 14C-labelled meta-tetrahydroxyphenylchlorin and 5-aminolaevulinic acid in the C6 rat glioma model Br J Cancer 78 733788 9743291 10.1038/bjc.1998.569 PMC2062980

[bib18] PougeBBurkeG1998 Fiber-optic bundle design for quantitative fluorescence measurements from tissue Appl Optics 37 74297436 10.1364/ao.37.007429 18301577

[bib19] RisHBAltermattHJNachburBStewartCMWangQLimCKBonnettRAlthausU1996 Intraoperative photodynamic therapy with m-tetrahydroxyphenylchlorin for chest malignancies Lasers Surg Med 18 3945 8850464 10.1002/(SICI)1096-9101(1996)18:1<39::AID-LSM5>3.0.CO;2-S

[bib20] SavaryJFMonnierPFontollietCMizeretJWagnieresGBraichotteDvan den BerghH1997 Photodynamic therapy for early squamous cell carcinomas of the esophagus, bronchi, and mouth with m-tetra (hydroxyphenyl) chlorin Arch Otolaryngol Head Neck Surg 123 162168 9046283 10.1001/archotol.1997.01900020042006

[bib21] TraunerKGandour-EdwardsRBambergMShortkoffSSledgeCHasanT1998 Photodynamic synovectomy using benzoporphyrin derivative in an antigen-induced model for rheumatoid arthritis Photochem Photobiol 67 133139 9477771

[bib22] WagnièresGStarWWilsonB1998 *In vivo* fluorescence spectroscopy and imaging for oncological applications Photochem Photobiol 68 603632 9825692

[bib23] WestermannPGlanzmannTAndrejevicSBraichotteDForrerMWagnièresGMonnierPvan den BerghHMachJ-PFolliS1998 Long circulating half-life and high tumour selectivity of the photosensitiser meta-tetrahydroxyphenylchlorin conjugated to polyethylene glycol in nude mice grafted with human colon carcinoma Int J Cancer 76 842850 9626351 10.1002/(sici)1097-0215(19980610)76:6<842::aid-ijc13>3.0.co;2-4

[bib24] WorkmanPBalmainAHickmanJAMcNallyNJMitchisonNAPierrepointCGRaymondRRowlattCStephensTCWallaceJ1998 United Kingdom co-ordinating committee on cancer of cancer research (UKCCCR) guidelines for the welfare of animals in experimental neoplasia(second edition)Br J Cancer 77 110 10.1038/bjc.1998.1 PMC2151254 9459138

[bib25] WhelptonRMichael-TitusATJamdarRPAbdillahiKGrahnMF1996 Distribution and excretion of radiolabeled temoporfin in a murine tumour Photochem Photobiol 63 885891 8992509 10.1111/j.1751-1097.1996.tb09646.x

[bib26] ZellwegerMGrosjeanPMonnierPvan den BerghHWagnièresG1999 Stability of the fluorescence measurement of Foscan in the normal human oral cavity as an indicator of its content in early cancers of the esophagus and the bronchi Photochem Photobiol 69 605610 10333768

